# Extracellular Disposal of Tumor-Suppressor miRs-145 and -34a via Microvesicles and 5-FU Resistance of Human Colon Cancer Cells

**DOI:** 10.3390/ijms15011392

**Published:** 2014-01-20

**Authors:** Yukihiro Akao, Fiona Khoo, Minami Kumazaki, Haruka Shinohara, Kohei Miki, Nami Yamada

**Affiliations:** United Graduate School of Drug Discovery and Medical Information Sciences, Gifu University, 1-1 Yanagido, Gifu 501-1193, Japan; E-Mails: fiona_khoo@ezweb.ne.jp (F.K.); r3125013@edu.gifu-u.ac.jp (M.K.); 086019@gifu-pu.ac.jp (H.S.); sur144@poh.osaka-med.ac.jp (K.M.); gif187@gifu-u.ac.jp (N.Y.)

**Keywords:** miR-34a, miR-145, 5-FU, microvesicles, drug resistance

## Abstract

The dysregulation of microRNA (miRNA) expression causes various kinds of diseases. Especially, alterations in miRNA expression levels are frequently observed in human tumor cells and are associated with cancer pathogenesis. Earlier we established Fluorouracil (5-FU)-resistant human colon cancer DLD-1 cells (DLD-1/5FU) from parental 5-FU- sensitive DLD-1 cells. In the present study, we examined the expression of miRNA in each cell line and in its extracellular microvesicles (MVs) before and after treatment with 5-FU. The nascent RNAs of anti-oncogenic miR-34a and -145 labeled with EU in both cells were proved to be transferred into MVs in both cell lines. The levels of miR-34a and -145 in the cells and in their MVs were not largely different in the two cell lines, and a substantial amount of both miRNAs was secreted by both cell lines even in the steady-state condition. The exposure of both cell lines to 5-FU significantly increased the intracellular levels of miR-145 and miR-34a in the 5-FU-sensitive DLD-1 cells, whereas the level of neither miR was elevated in the DLD-1/5FU cells. Interestingly, the amount of miR-145 detected in the small MVs shed into the medium of the parental cells was reduced after the treatment with 5-FU. On the other hand, the intracellular expression of miR-34a in the DLD-1/5FU cells was down-regulated compared with that in the parental DLD-1 cells even in the steady-state condition. As to the miR-34a secreted into MVs, the increase in the level in DLD-1/5FU cells was greater than that in the parental DLD-1 cells after the treatment with 5-FU. Thus, the intra- and extracellular miR-145 and -34a were closely associated with 5-FU resistance, and the resistance was in part due to the enhanced secretion of miR-145 and -34a via MVs, resulting in low intracellular levels of both miRNAs.

## 1. Introduction

MicroRNAs (miRNAs) are non-coding RNAs that negatively regulate translation by imperfectly binding to complementary target messenger RNAs (mRNAs). Over the last decade, approximately 1500 human miRNAs have been identified (miRBase; http://www.mirbase.org/), and implicated in the regulation of a wide range of cellular processes, including differentiation, proliferation, and apoptosis. The dysregulation of miRNA expression causes various kinds of diseases. Especially, alterations in expression levels of miRNAs are frequently observed in human tumor cells and are associated with cancer pathogenesis. In human colon tumors, we demonstrated earlier that miR-143 and -145, and -34a are down-regulated in most adenomas and cancers compared with their levels in non-tumorous tissues in the same patients [1]. Our previous reports and others also indicate that these miRNAs function as anti-oncomirs [1–10]. These miRNAs are major anti-oncomirs in colon cancer cells. The up-regulation of miR-143 and -145 by the phytochemical α-mangostin causes growth inhibition through down-regulation of *Erk5* and *c-Myc*, which are known to be target mRNAs of miR-143 and -145, respectively [11]. Also, we recently reported that resveratrol up-regulates miR-34a, which triggers the miR-34a/E2F3/Sirt1 cascade and contributes to the induction of apoptosis by resveratrol in colon cancer DLD-1 cells [12].

Previously, we reported that the level of miR-34a is significantly down-regulated in 5-FU-resistant DLD-1 (DLD-1/5FU) cells compared with that in the parental 5-FU-sensitive DLD-1 cells [13]. Additionally, the exposure of both cell lines to 5-FU elevates the intracellular level of miR-34a level in the parental cells; whereas the level in DLD-1/5FU cells remains almost unchanged. The increased miR-34a level in the parental cells suppresses the expression of its target genes *Sirt1* and *E2F*, thus inducing growth inhibition in DLD-1 cells [13]. On the other hand, much evidence revealed that the expression levels of miR-143 and -145 are markedly down-regulated in various kinds of gastrointestinal tumors [2,6,9] and other tumors such as B-cell lymphomas [3], mammary cancers [14], and nasal-pharyngeal cancers [15]. It has been considered that the genomic abnormality, epigenetic changes, aberrant transcription, and impaired processing of miRNA causes this low expression of anti-oncomirs. MiR-34a is well known to function as a cell-death inducer by targeting key molecules that protect against apoptosis [12,16,17]. In this present study, we demonstrated that miR-145 and -34a are released in response to 5-FU exposure as passengers in microvesicles (MVs) consisting of shedding microvesicles (SMVs) and exosomes (EXOs), which are defined as secretory miRNAs [18–20], probably to maintain the intracellular low levels of these miRs in human colon cancer DLD-1 cells. This machinery may contribute to the maintenance of cancer cell growth and resistance to anti-cancer drugs.

## 2. Results

**Figure 1.**
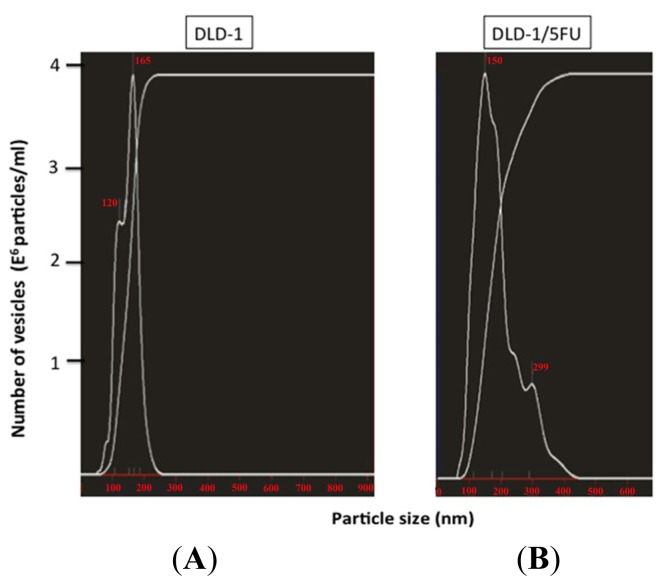
Characteristics of microvesicles from human colon cancer DLD-1 cells as determined by Nanoparticle Tracking Analysis (NTA). MVs were isolated by centrifugation and filtered through the 0.22-μm filter of the ExoMir kit. The Nanosight LM10 nanoparticle characterization system (NanoSight, NanoSight Ltd., Amesbury, UK) equipped with blue-laser (638 nm) illumination was used for real-time characterization of the vesicles. The results are presented at the average value of two independent experiments in human colon cancer DLD-1 (**A**) and 5-FU resistant DLD-1/5FU (**B**) cells. The number of MVs (E6 particles/mL) and the size distribution (particle diameter, nm) are shown on the *y* axis and *x* axis, respectively.

**Figure 2.**
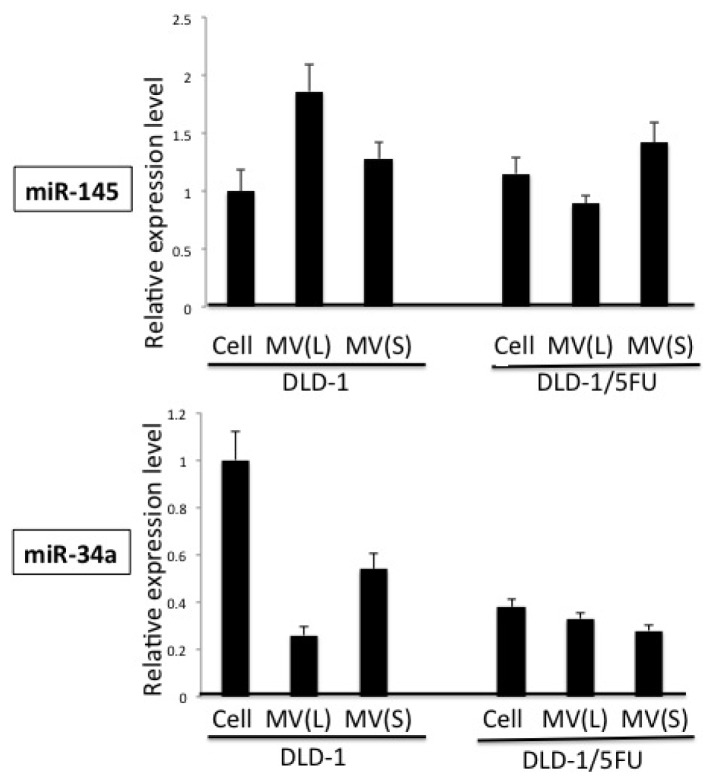
Tracing EU-labeled miR-145 and -34a from donor cells into MVs. Amounts of EU-labeled nascent miR-145 and -34a in DLD-1 and DLD-1/5FU cells and in their secreted MVs are shown. The relative quantities of EU-labeled miRNA are given, with the amount of the expression in DLD-1 cells indicated as “1”. The results are presented as the average value of three independent experiments. MV(L) and MV(S) are MVs filtered through the 1st filter and 2nd filter, respectively, of the ExoMir kit.

**Figure 3.**
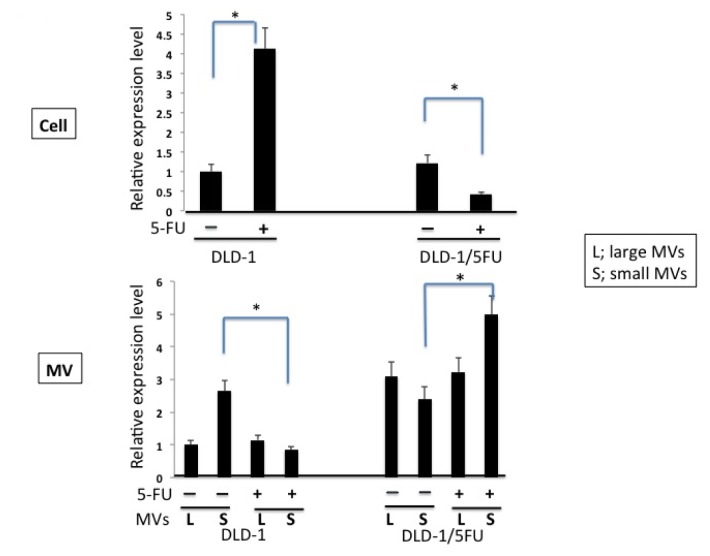
Detection of miR-145 levels in DLD-1 and DLD-1/5FU cells and in their MVs. Levels of miR-145 in the cells (the upper panel) and in their MVs (the lower panel) trapped by the 1st (L) and 2nd (S) filters of the ExoMir kit. The upper panel shows the relative expression of miRNA in the cells before and after the treatment with 5-FU (10 μM) for 48 h; and the relative quantities are given relative to the amount in DLD-1 cells indicated as “1”, as in the case of MVs. The results are presented as the average value of three independent experiments. MV(L) and MV(S) were MVs filtered through 1st filter and 2nd filter, respectively, of the ExoMir kit. “+”: treatment with 5-FU; “−”: treatment with DMSO alone. * *p* < 0.05 is considered to be significant.

Earlier we reported that the levels of miRs-143, -145, and -34a are extremely down-regulated in human colon cancer tissues and cell lines, and that they function as anti-oncomirs [1]. Notably, the level of miR-34a in 5-FU-resistant DLD-1/5FU cells is extremely low compared to that in the parental DLD-1 cells [13]. Also, the level of miR-34a in DLD-1 cells, but not that in DLD-1/5FU cells, is increased after the exposure to 5-FU for 48 h. Such a difference in response between DLD-1 and DLD-1/5FU cells could mainly contribute to the resistance to 5-FU, which is elicited by the miR-34a/target gene cascade [13,16,17]. In regard to the machinery responsible for the down-regulation of anti-oncomirs in cancer cells, there is a possibility that cancer cells eliminate anti-oncomirs by secreting them via MVs. In order to validate such a possibility, we examined the levels of miR-145 and -34a between MVs and the cells to clarify whether the released MV-entrapped miRNA (MV/miR) was associated with the maintenance of cancer cell growth and drug resistance. As to isolation of MVs from the culture medium, we had already established a centrifugation/filtration method using an ExoMir kit, and we validated the character of MVs secreted from DLD-1 cells by the NTA method (Figure 1) [19,20]. The NTA analysis indicated that the population of MVs longer than 200 nM in diameter was increased in DLD-1/5FU cells, compared with that in DLD-1 cells. In order to prove the secretion of cellular miRNAs via MVs, we examined the processing of EU-labeled nascent RNAs. The intracellular EU-labeled RNAs surely moved into MVs; this movement was estimated by qRT-PCR using real-time PCR (Figure 2). Importantly, both cell lines secreted the nascent miR-145 and -34a to some extent as passengers in MVs. However, a significant amount of the nascent miR-143 was not detected in MVs. We defined such MV/miRNAs as secreted miRNAs. The levels of miR-145 in both cell lines were almost the same. The level of intracellular miRNA-145 in DLD-1 cells was significantly increased after the exposure to 5-FU; however, that in the DLD-1/5FU cells was markedly decreased, accompanied by significant secretion of miR-145 via MVs (Figure 3). At the same time, the miR-145 released via the small MVs was remarkably down-regulated in the DLD-1 cells. These results suggest that the level of intracellular miR-145 was elevated possibly due to the perturbed secretion of miR-145 in response to 5-FU exposure. On the other hand, DLD-1/5FU cells consistently secreted miR-145 to maintain the intracellular low-levels of miR-145 compared with DLD-1 cells, which secretion was not affected by the 5-FU exposure.

**Figure 4.**
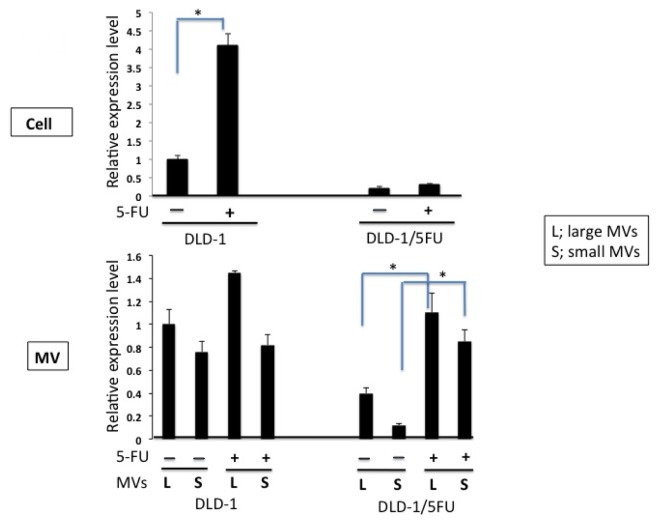
Detection of miR-34a levels in DLD-1 and DLD-1/5FU cells and in their MVs. Levels of miR-34a in the cells and in the MVs trapped by the two filters of the ExoMir kit. The upper panel shows the relative expression of miRNAs before and after the treatment with 5-FU (10 μM) for 48 h, with the quantities given being relative to the amount in DLD-1 cells indicated as “1”. Lower panel shows the relative expression of the miRNAs in MVs from DLD-1 and DLD-1/5FU cells before and after the treatment with 5-FU (10 μM) for 48 h. The results are presented at the average value of three independent experiments. “+”: treatment with 5-FU; “−”: treatment with DMSO alone.* *p* < 0.05 is considered to be significant.

As to miR-34a, the level of intracellular miR-34a in DLD-1/5FU cells was strikingly lower than that in DLD-1 cells (Figure 4), as reported previously by us [13]. On the other hand, the level of intracellular miR-34a in DLD-1 cells was significantly increased in response to 5-FU exposure, an effect that was not observed in DLD-1/5FU cells. The level of miR-34a in the large MVs from DLD-1 cells tended to be increased, but the increase was not significant enough to avoid cell death due to exposure to 5-FU. Notably, the miR-34a levels in the large and small MVs from the 5-FU-resistant cells were markedly elevated by 3–4 fold in response to 5-FU exposure, resulting in the maintenance of a low intracellular level.

## 3. Discussion

**Figure 5.**
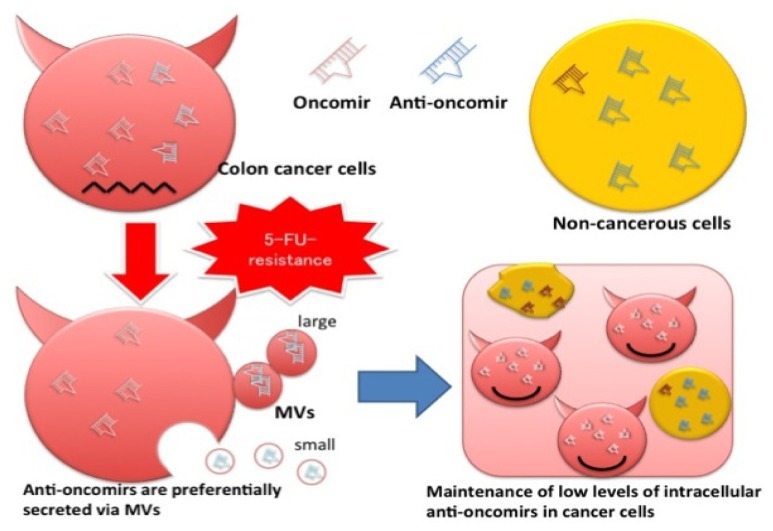
Schematic diagram of machinery for drug resistance in DLD-1/5FU cells.

We had already established the MV collection method for samples such as culture medium [19,20] and blood [20,21], which were evaluated by NTA and biochemical analysis. In the current study, we demonstrated that the miR-145 and -34a, which are well-known anti-oncomirs in colon cancer cells, were positively secreted via MVs, because of the substantial amounts of both miRNAs in MVs compared with those in the cells and that their levels in both cells and MVs were affected by 5-FU exposure. The size distribution of MVs from DLD-1/5FU cells was significantly different from that of DLD-1 cells. The population of the large MVs was increased, which indicated that SMVs in DLD-1/5FU cells were more than those in DLD-1 cells. The results of the Click-iT nascent RNA capture assay clearly indicated that the neo-generated RNA molecules of the miRNAs in the both cell lines are secreted in part via MVs. The levels of intracellular miRs-145 and -34a in the DLD-1 cells were significantly increased and the release of MV/miR-145 from these cells was perturbed by 5-FU treatment, which could be one of the mechanisms responsible for the inhibition of cell growth by 5-FU. In contrast, the 5-FU-resistant DLD-1/5FU cells showed a reduced intracellular miR-145 level possibly due to increased secretion of miRs-145 and -34a via MVs after 5-FU exposure. Similarly, the intracellular levels of miR-34a in DLD-1 cells were 3- to 4-fold up-regulated compared with that in 5-FU-resistant cells. The levels of miR-34a secreted via MVs were similar in both types of cells, though different from those of miR-145. Thus, in response to 5-FU exposure, the changes in miRNA expression and secretion via MVs were different between 5-FU-sensitive and -resistant cells, indicating an association between MV secretion and resistance (Figure 5). MiR-34a is closely associated with 5-FU resistance [13,22]. In order to clarify the relationship between MV secretion of anti-oncomirs and the resistance to 5-FU, we treated DLD-1/5FU cells with neutral sphingomyelinase-2 inhibitor that was reported to perturb the secretion of exosome [23]. However, we found no difference in the numbers of the secreted MVs between treated and non-treated cells by NTA. It needs to be clarified which signal pathways are involved in the selective secretion of miRNAs via MVs and in their machineries, and also the roles of miRs-34a and -145 within the MVs in cell-to-cell communication. In the future, molecules identified to prevent this selective secretion via MVs will become novel targets for the development of anti-cancer drugs.

## 4. Experimental Section

### 4.1. Cell Lines

The 5-FU-sensitive human colon cancer cell line DLD-1 was purchased from JCRB (Osaka, Japan). A 5-FU resistant DLD-1/5FU cell line [13] was obtained from DLD-1 cells after selection by drug exposure. They were cultured in RPMI 1640 medium supplemented with 10% (*v*/*v*) heat-inactivated fetal bovine serum (Sigma, St. Louis, MO, USA) under an atmosphere of 95% air and 5% CO_2_ at 37 °C. 5-FU was obtained from Sigma (St. Louis, MO, USA) and dissolved in Dimethyl sulfoxide (DMSO). The *IC*_50_ values in DLD-1 and DLD-1/5FU cells for 5-FU are 3.9 and 38.8 μM, respectively.

### 4.2. Collection of MVs from the Supernatant of Culture Medium

For exclusion of calf-derived MVs from fetal calf serum (FCS), the serum was centrifuged at 250,000*g* for 3 h (Beckman Coulter, Inc., Fullerton, CA, USA) and then the supernatant was further passed through a 0.45-μm filter [20,21]. The filtered serum was used in all experiments. The cells were treated or not treated with 5-FU at the concentration of 10 μM for 48 h in calf MV-free 10% FCS medium; and then the MVs shed from the cells into the medium were collected. In brief, the MV-containing medium was firstly centrifuged at a low-grade speed (3000 rpm). Then, the resulting supernatant was further ultracentrifuged (250,000*g* for 3 h), after which the pellet was resuspended in PBS. This suspension was then filtered through 2 filters of an ExoMir kit™ (BiooSicentific; Austin, TX, USA): through the 1st filter (0.22 μm) to obtain large MVs (MVs(L)) and through the 2nd filter (0.02 μm) for the small MVs (MVs(S)). It seemed that the large MVs contain SMVs and EXOs, and the small MVs contain mainly EXOs [18].

### 4.3. Nanoparticle Tracking Analysis (NTA)

MVs were purified from DLD-1 and DLD-1/5FU cells. The procedure is described in supplementary Figure S1. The MVs after passage through the 1st filter of the ExoMir kit (BiooSicentific; Austin, TX, USA) were used for analysis. The Nanosight LM10 nanoparticle characterization system (NanoSight, Amesbury, UK) equipped with a blue laser (638 nm) for illumination was used for real-time characterization of the vesicles. The results were presented at the average value of two independent experiments. The number of MVs (E6 particles/mL) and the size distribution (particle diameter, nm) are shown on the *y* axis and *x* axis, respectively.

### 4.4. RNA Extraction from Microvesicles

For RNA extraction from MVs prepared by use of the ExoMir kit (BiooSicentific; Austin, TX, USA), the MV-bearing 1st and 2nd filters were disconnected and then separately flushed with BiooPure-MP (BiooSicentific, Austin, TX, USA) to lyse the captured particles and to release their contents. BiooPure-MP (BiooSicentific, Austin, TX, USA) is a single-phase RNA extraction reagent containing guanidinium thiocyanate and phenol, which has been optimized to provide maximal recovery of the low-mass amounts of RNA in MVs.

### 4.5. Quantitative RT-PCR Using Real-Time PCR

To determine the expression of miRNAs, we measured their levels by using TaqMan® (Applied Biosystems, Foster City, CA, USA) miRNA assays, which included RT primers and TaqMan probes (Applied Biosystems, Foster City, CA, USA). The PCR procedure was performed by real-time PCR. Briefly, after reverse transcription of 25 ng of total-RNA, cDNA was generated. The PCR reaction consisted of 40 cycles (95 °C for 5 s, 60 °C for 30 s) after an initial denaturation step (95 °C for 10 s). Expression levels of microRNAs were detected by using a TaqMan microRNA reverse transcription kit and TaqMan microRNA Assay Kit (Applied Biosystems, Foster City, CA, USA) according to the manufacturer’s protocol. *RNU6B* was used for an internal standard. Calculation of the *C*t value was done by using the second-derivative maximum method, and relative quantification was analyzed by performing comparative *C*t (ΔΔ*C*t). All reactions were run in triplicate. *MiR-21* levels were used as internal controls for the cells and MVs (the mean *C*t value: 25.84 (Cells); 30.29 (MVs)), respectively. We purified prepared total RNA samples by use of an RNeasy mini kit (Qiagen, Hilden, Germany); and the total-RNA was reverse-transcribed with a SuperScript^™^ III kit (Invitrogen, Carlsbad, CA, USA).

### 4.6. Study of RNA Synthesis, Transcriptional Regulation, and Secretion of Newly Synthesized RNA Molecules

Living cells were incubated with an analog of uridine, 5-ethynyl uridine (EU) (Invitrogen, Carlsbad, CA, USA), which is efficiently and naturally incorporated into nascent RNA. After the incubation, total RNA labeled with an appropriate volume of 200 mM EU was isolated and used in a copper catalyzed click reaction with an azide-modified biotin, which creates a biotin-based handle for capturing nascent RNA transcripts on streptavidin magnetic beads. Then, the captured transcripts were used as a template for reverse transcriptase-mediated cDNA synthesis for subsequent analysis using qPCR. EU has been shown to be non-toxic to the cells as evidenced by growth curves (data not shown).

### 4.7. Statistics

Differences were statistically evaluated by using student’s *t*-test. A *p*-value of less than 0.05 was considered to be statistically significant.

## 5. Conclusions

In conclusion, human colon cancer cells preferentially secreted anti-oncomirs such as miR-34a and miR-145 via microvesicles in response to 5-FU exposure, resulting in decrease in the intracellular levels of the anti-oncomirs. This finding indicates one of the mechanisms for drug resistance and maintenance of proliferation in cancer cells.

## Supplementary Information
