# Supplementary material for: Extracellular Disposal of Tumor-Suppressor miRs-145 and -34a via Microvesicles and 5-FU Resistance of Human Colon Cancer Cells
Source: Int J Mol Sci. 2014 Jan 20;15(1):1392–401. doi: 10.3390/ijms15011392 (PMC3907875; doi:10.3390/ijms15011392)
Supplement: Supplementary file 1 [file ijms-15-01392-s001.pdf]

Supplementary Information

Figure S1. Method of MVs isolation.

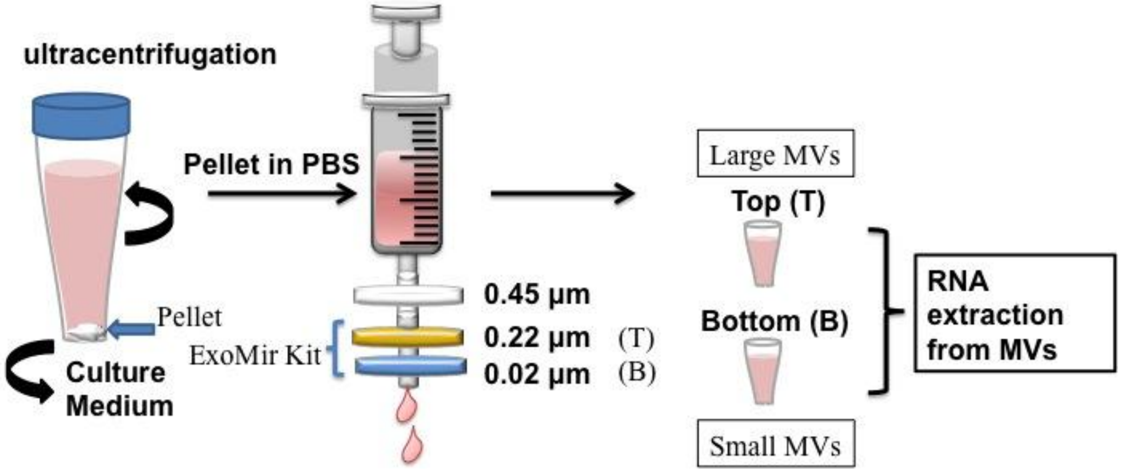

© 2014 by the authors; licensee MDPI, Basel, Switzerland. This article is an open access article distributed under the terms and conditions of the Creative Commons Attribution license (http://creativecommons.org/licenses/by/3.0/).
